# Evaluation of factors that influenced the length of hospital stay using data mining techniques

**DOI:** 10.1186/s12911-022-02027-w

**Published:** 2022-10-29

**Authors:** Mehrnoosh Eskandari, Amir Hossein Alizadeh Bahmani, Heydar Ali Mardani-Fard, Iman Karimzadeh, Navid Omidifar, Payam Peymani

**Affiliations:** 1 Health Policy Research Center, Institute of Health, Shiraz University of Medical Sciences, Shiraz, Iran; 2 Department of Pulmonary Medicine, Amsterdam UMC location University of Amsterdam, Meibergdreef 9, Amsterdam, the Netherlands; 3 Department of Mathematics, Yasouj University, Yasouj, Iran; 4 Clinical Pharmacy Department, Faculty of Pharmacy, Shiraz University of Medical Sciences, Shiraz, Iran; 5 Department of Pathology, medical school, Shiraz University of Medical Sciences, Shiraz, Iran; 6 College of Pharmacy, Rady Faculty of Health Sciences, University of Manitoba, Apotex Centre, 750 McDermot Avenue, Winnipeg, MB R3E 0T5 Canada

**Keywords:** Length of stay, Hospitalization, Data mining, Tree plot

## Abstract

**Background:**

length of stay (LOS) is the time between hospital admission and discharge. LOS has an impact on hospital management and hospital care functions.

**Methods:**

A descriptive, retrospective study was designed on about 27,500 inpatients between March 2019 and 2020. Required data were collected from six wards (CCU, ICU, NICU, General, Maternity, and Women) in a teaching hospital. Clinical data such as demographic characteristics (age, sex), type of ward, and duration of hospital stay were analyzed by the R-studio program. Violin plots, bar charts, mosaic plots, and tree-based models were used to demonstrate the results.

**Results:**

The mean age of the population was 40.8 ± 19.2 years. The LOS of the study population was 2.43 ± 4.13 days. About 60% of patients were discharged after staying one day in the hospital. After staying one day in the hospital, 67% of women were discharged. However, 23% of men were discharged within this time frame. The majority of LOS in the CCU, ICU, and NICU ranged from 5 to 9 days.; In contrast, LOS was one day in General, Maternity, and Woman wards. Due to the tree plot, there was a different LOS pattern between Maternity-Women and the CCU-General-ICU-NICU wards group.

**Conclusion:**

We observed that patients with more severe diseases hospitalized in critical care wards had a longer LOS than those not admitted to critical care wards. The older patient had longer hospital LOS than the younger. By excluding Maternity and Woman wards, LOS in the hospital was comparable between males and females and demonstrated a similar pattern.

## Background

Inpatients’ length of stay (LOS) is defined as the time between hospital admission and discharge. It is an essential factor for hospitals. LOS has an impact on hospital management and hospital care functions. Because each hospital has a limited number of beds, staff, and care services in each ward, predicting LOS is critical in order to provide the best hospital services to inpatients. LOS, on the other hand, influences the number of hospital employees, beds, treatment outcomes, hospitalization fees, and hospital resource management [1, 2]. Hospitals and health care providers prefer shorter LOS to longer LOS. Decreasing LOS can decrease medication side effects, risk of hospital infection, and hospitalization costs for both patients and hospitals [3, 4]. LOS can be used to indicate difficult-to-measure research outcomes such as mortality and disease severity [5–8]. In terms of patient flow, LOS is critical. Patient flow represents how a patient moves through a sequence of procedures [9, 10]. LOS reduction and the conviction that each patient receives the appropriate care at the appropriate time have an impact on service quality, patient outcomes, and care costs. The payment pressure on governments and health institutions is directly impacted by LOS reduction [9].

Data mining is the process of extracting and discovering potentially valuable knowledge and information from a large amount of raw, noisy, fuzzily, and randomly generated practical data. Due to its excellent performance in assessing patient risks and assisting clinical decision-making when developing disease-prediction models, data mining technology has been a frontier area in medical research. Data mining models are classified into two types: descriptive and predictive. Descriptive models are mainly used to discover the data-describing patterns, whereas predictive models are used to estimate future parameters of interesting variables. Machine learning, statistics, database technology, and pattern recognition come together in the multidisciplinary field of data mining, which benefits from all of these disciplines [11, 12].

Decision trees and tree models are common data mining and clinical decision-making tools. In a tree model, at each step, the total population or upstream population splits into smaller subgroups with the highest different probability of an interesting outcome. Hence, a successful tree model can split the population into clusters with high between-cluster variability, but low within-cluster variability in the likelihood of an interesting outcome [13].

This study aims to evaluate the factors that may associate with the length of stay (LOS) in six different wards of a teaching hospital in Iran using statistical analysis along with a data mining technique by the R-studio program. We also used a decision tree model to cluster the studied population optimally.

## Methods

### Study setting and sampling

A descriptive, retrospective study was designed at Zeinabiyeh hospital, affiliated with Shiraz University of Medical Sciences, Shiraz, Iran, between March 2019 and March 2020. Qualitative and quantitative data were collected using HIS system and observations from six different wards (CCU, ICU, NICU, General, Maternity, and Women), including about 27,500 inpatients. The study was approved by the Medical Ethics Committee of Shiraz University of Medical Sciences (ethical ID: 17,541). The Helsinki declaration has been followed for all methods. The need for informed consent was waived by the ethics committee of Shiraz University of Medical Sciences because of the retrospective nature of the study.

### Data preprocessing

In order to data mining, clinical data were analyzed by the R software 4.1.1 (2021-08-10) and R Studio 2021.09.0 + 351. Violin plots, bar charts, mosaic plots, and tree-based models were used to demonstrate the results. In this work, underlying variables were the number of hospitalization days varying between 0 and 300 days, the gender of the patients, the age of the patients, and the ward where the patient is hospitalized. These data were extracted from the automated Hospital Information System (HIS).

### Tree model

The classification tree method is a decision tree-based model for analyzing data with categorical response variables. The decision tree’s ultimate goal is to divide the observations into several groups based on auxiliary variables to have the least within-group and the most between-group differences in the response variable.

The decision tree method works hierarchically. Each stage selects one of the previously created groups and divides it into two subgroups based on one of the auxiliary variables.

The selection of such group and the auxiliary variable, as well as how to divide the values ​​of the selected auxiliary variable is done based on a series of specific criteria (Akaike Information Criterion (AIC), Bayesian Information Criterion (BIC), Mallows’s Cp, R-squared, and adjusted R-squared) so that we get as close as possible to the final goal.

This algorithm is usually stopped based on certain criteria such as maximum tree depth, the minimum number of observations in the end nodes, and some statistical criteria such as the ratio of the within-groups sum of squares of deviations to the between-groups sum of squares of deviations. In each end node, we can either estimate the probability mass of the response variable (as the relative frequency of the levels of the response variable) or assign the whole observations of that end node to the response variable’s level with the most relative frequency. More precisely, if ${Y}_{1},\dots , {Y}_{d}$ be the levels of the response variable, $y$, then the probability mass of $y$ in the $j$^th^ end node is estimated by:

$${P}_{j}\left[{Y}_{i}\right]\approx \frac{\#\left\{{y}_{jk}\in {Y}_{i}\right\}}{{n}_{j}},$$

where ${y}_{jk}$, $k=1, 2, \dots , {n}_{j}$ be the $k$th observations of the $j$th end node, and ${n}_{j}$ is the number of observations [14].

In this study, to predict the hospitalization time, the hospitalization time is categorized into eight categories: 0 days, one day, two days, 3–4 days, 5–9, 10–14 days, 15–24 days, and greater than 25 days. A tree model is then fitted with this categorized variable as response and gender, age, and ward as the auxiliary variables.

Categorical variables were expressed as a percentage. The normal distribution of continuous variables was examined using the Kolmogorov-Smirnov test. Continuous data with and without a normal distribution were expressed as mean ± SD and median (interquartile range), respectively.

## Results

Totally 27,505 inpatients in six different wards and their data were included in this study. The mean ± SD age of the population was 40.8 ± 19.2 years. More than three-fourths of the study population (85.2%) were females, and the remaining 4074 (14.8%) were males (Table 1). The distribution of patients in CCU, General, ICU, Maternity, NICU and Women wards were 2430 (8.8%), 6740 (24.5%), 1167 (4.2%), 7894 (28.7%), 556 (2%), and 8718 (31.7%), respectively (Table 2). The mean ± SD LOS of the study population was 2.43 ± 4.13 days.

**Table 1:** The frequency of LOS for female and male patients stayed in the hospital. LOS: length of stay

Stay (day)	Female	Male	Total (% of total patients)	
0	886	139	1025	(3.7%)
1	15,622	951	16,573	(60.3%)
2	2677	704	3381	(12.3%)
3–4	2035	889	2924	(10.6%)
5–9	1596	1009	2605	(9.5%)
10–14	374	221	595	(2.2%)
15–24	154	102	256	(0.9%)
25+	87	59	146	(0.5%)
Total (% of total patients)	23,431 (85.2%)	4074 (14.8%)	27,505 (100%)

**Table 2:** The frequency and percentage of patients stayed in each ward. *The proportion of each ward to the total population

Stay (day)	Ward											
	CCU (% of ward)		General (% of ward)		ICU (% of ward)		Maternity (% of ward)		NICU (% of ward)		Women (% of ward)	
0	43	(1.8%)	486	(7.2%)	10	(0.9%)	155	(2%)	11	(2%)	320	(3.7%)
1	317	(13%)	2110	(31.3%)	81	(6.9%)	7011	(88.9%)	63	(11.3%)	6975	(80.1%)
2	372	(15.3%)	1251	(18.6%)	113	(9.7%)	583	(7.4%)	67	(12.1%)	994	(11.4%)
3–4	729	(30%)	1347	(20%)	279	(23.9%)	93	(1.2%)	108	(19.4%)	368	(4.2%)
5–9	760	(31.3%)	1182	(17.5%)	374	(32%)	32	(0.4%)	207	(37.2%)	47	(0.5%)
10–14	141	(5.8%)	234	(3.5%)	151	(12.9%)	1	(0%)	65	(11.7%)	2	(0%)
15–24	47	(1.9%)	85	(1.3%)	101	(8.7%)	2	(0.1%)	18	(3.2%)	1	(0%)
25+	21	(0.9%)	45	(0.7%)	58	(5%)	5	(0.1%)	17	(3.1%)	0	(0%)
Total	2430	(100%)	6740	(100%)	1167	(100%)	7894	(100%)	556	(100%)	8718	(100%)
% of total patients^*^	8.8%		24.5%		4.2%		28.7%		2.0%		31.7%	

**Fig. 1:**
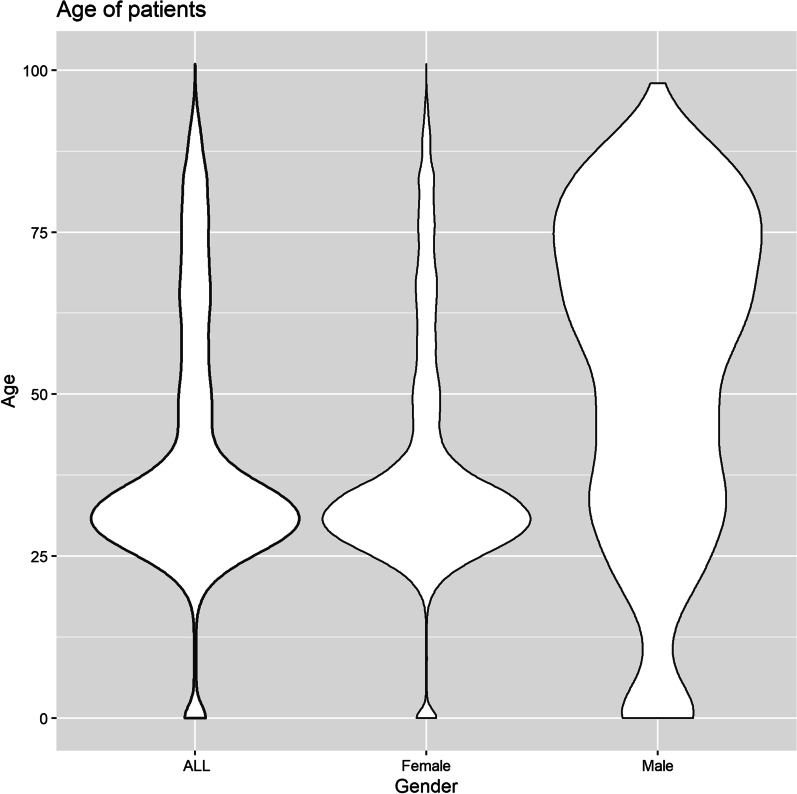
Violin plot that shows the dispersion of patients’ age in two different groups; men and women

**Fig. 2:**
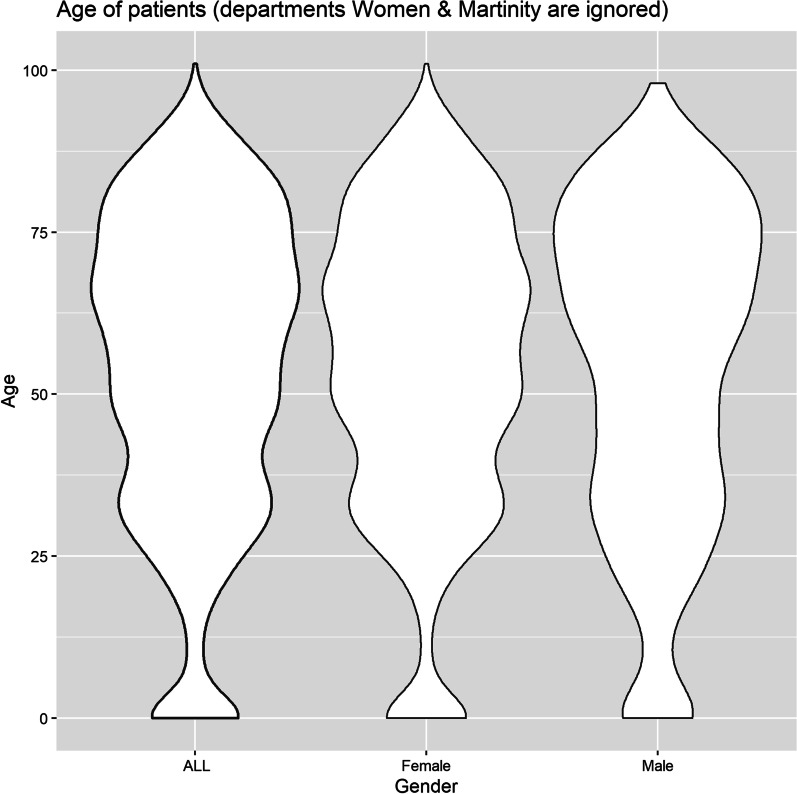
Violin plot demonstrates the distribution of age of patients who stayed in the hospital except for Women and Maternity wards in two different groups; women and men

**Fig. 3:**
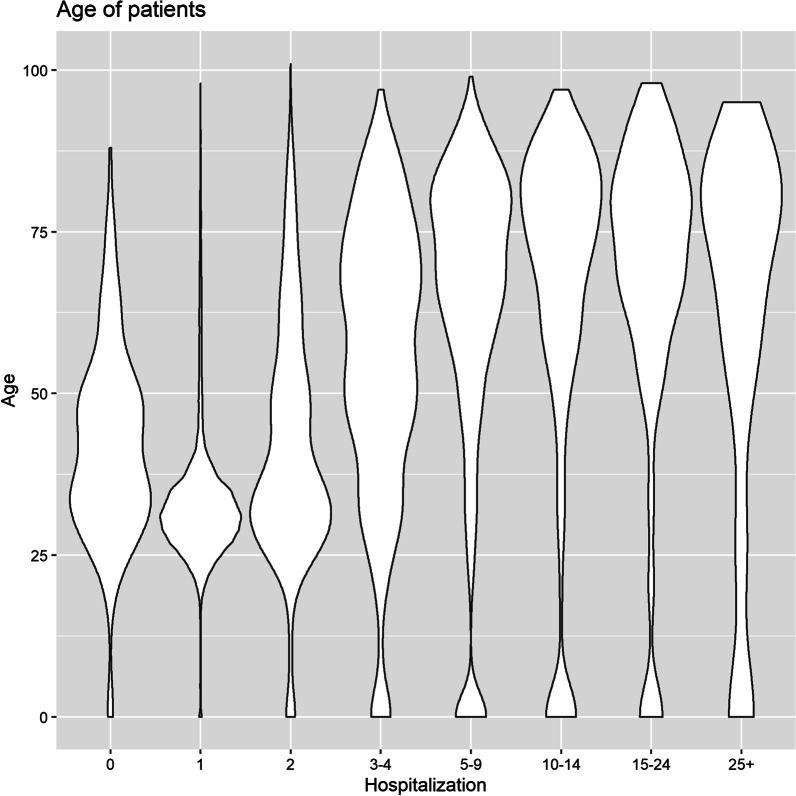
Violin plot indicates the dispersion of the age of patients with different duration of hospitalization

**Fig. 4:**
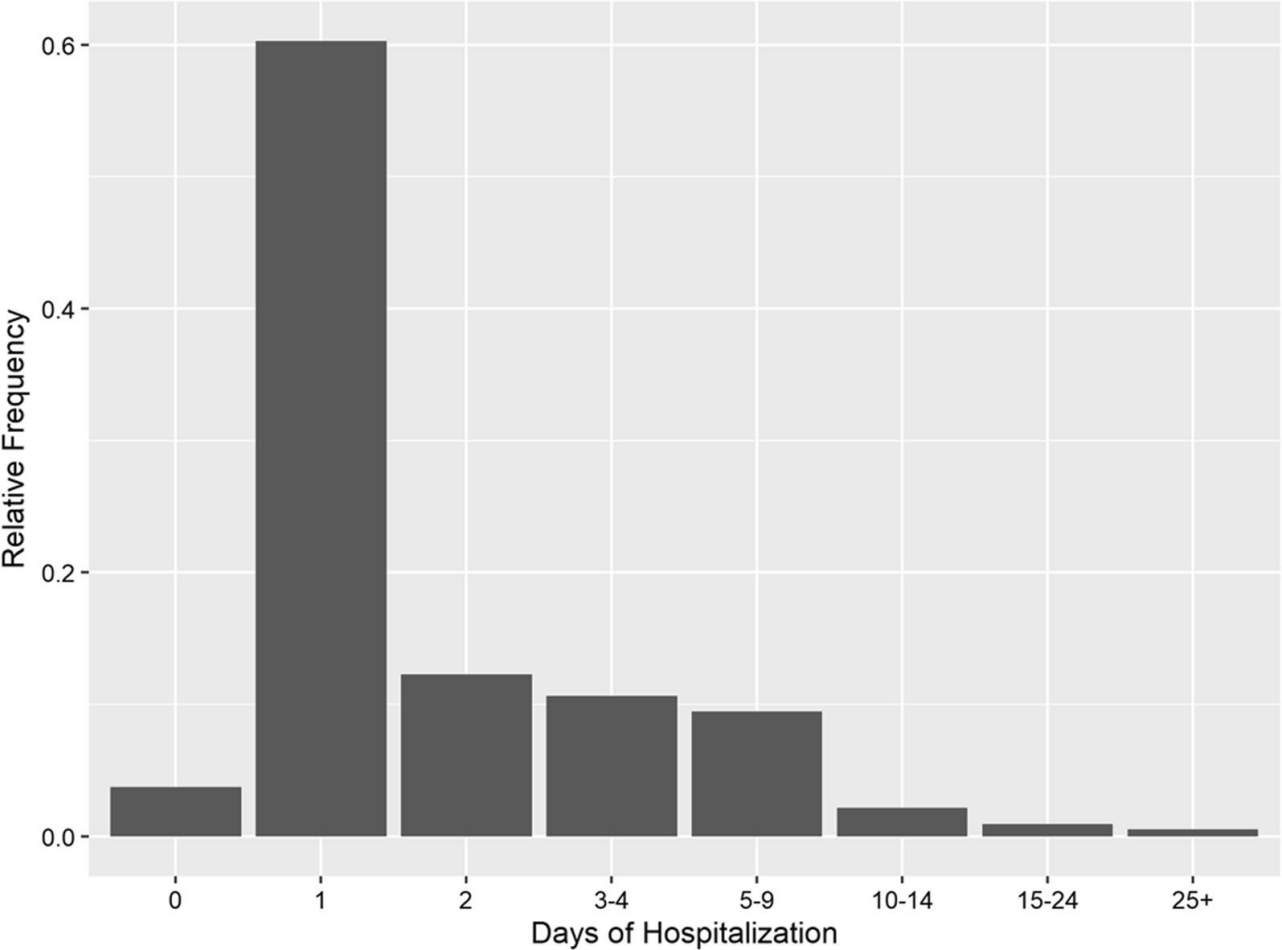
Bar chart that shows days of hospitalization of patients

**Fig. 5:**
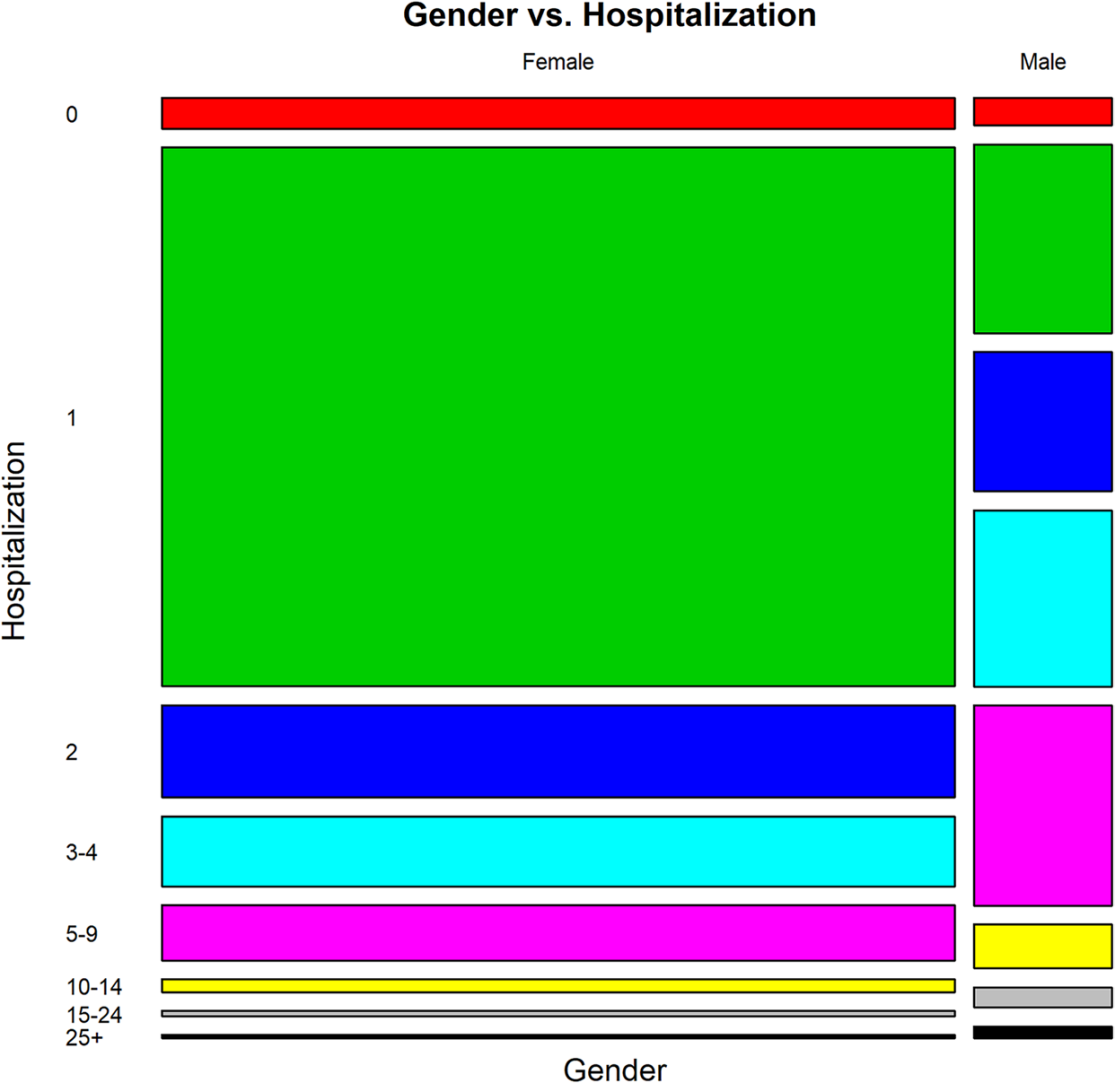
Mosaic plot that indicates the gender of patients versus days of hospitalization

**Fig. 6:**
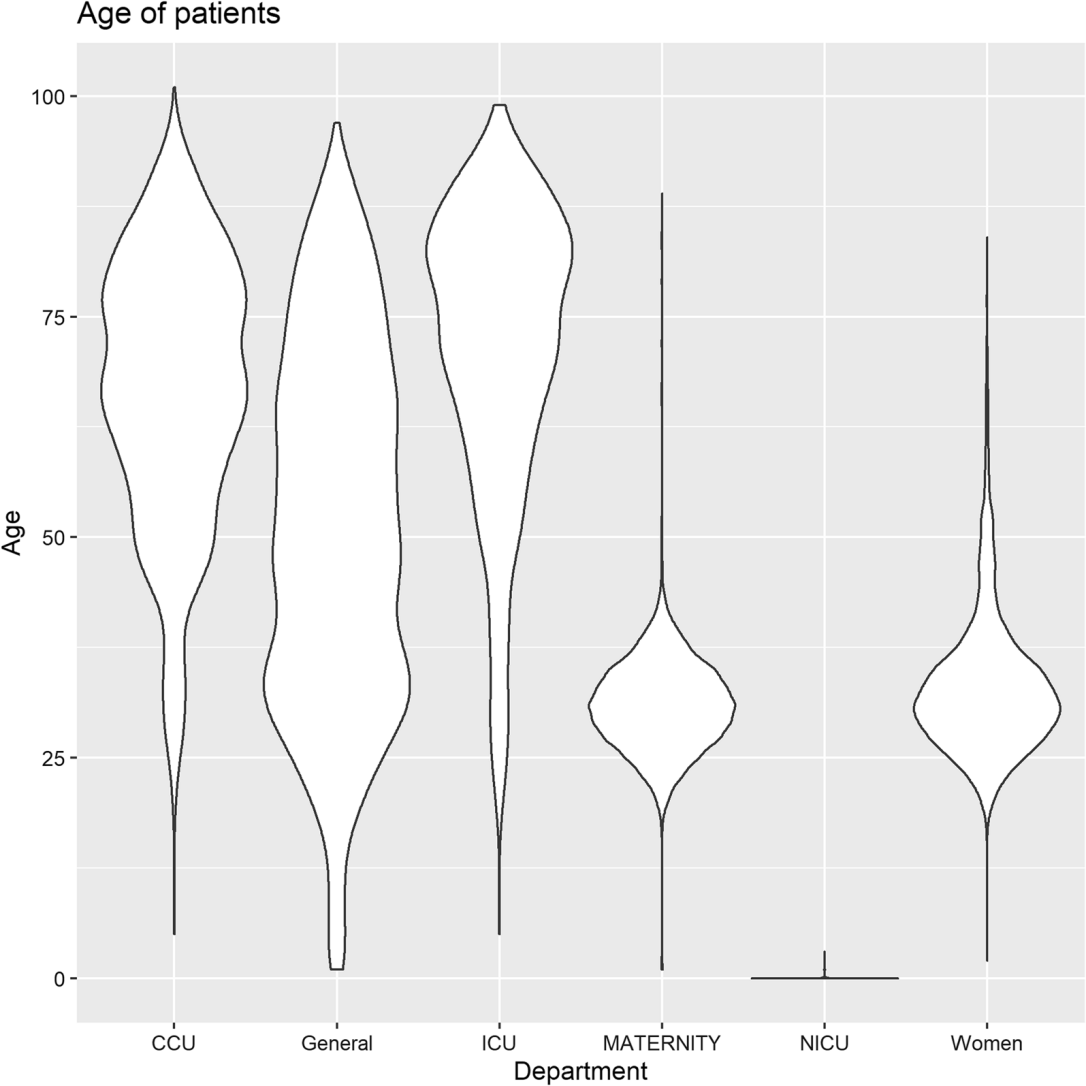
Violin plot shows the distribution of the age of patients who stayed in different wards

**Fig. 7:**
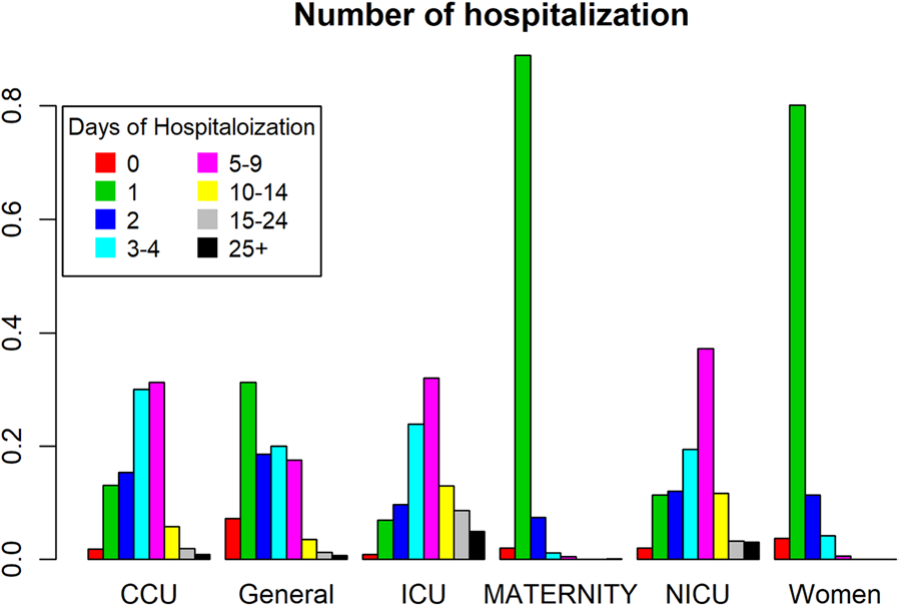
Bar chart that indicates days of hospitalization in different wards

The findings showed that older patients stayed in the hospital for a longer period (Figs. 1 and 2). However, participants had a wide range of ages discharged on the first day of hospitalization (Fig. 3). About 60% of patients were discharged after spending one night in the hospital (Fig. 4). About 67% of women were discharged after one day of hospitalization. However, 23% of men were discharged within this duration. Men usually showed longer LOS than women (Figs. 1 and 5). About 7011 (89%) and 6975 (80%) of females in the Maternity and Women wards were discharged after one day of hospitalization, respectively (Table 3). The biggest group of LOS in CCU, ICU, and NICU was 5–9 days; In contrast, in General, Maternity, and Woman wards, it was one day (Table 2; Figs. 6 and 7). The majority of patients who remained in CCU and ICU were older than 50; Even ICU patients were older than CCU patients on average. In contrast, most of the patients in maternity and women’s wards were between the ages of 22 and 37. On the other hand, patients who stayed in the general ward ranged in age from 13 to over 80 (Fig. 6).

**Table 3:** The frequency of female and male patients stayed in different wards

Stay (day)	Female						Male					
	CCU	General	ICU	Maternity	NICU	Women	CCU	General	Maternity	ICU	NICU	Women
0	19	385	2	155	5	320	24	101	0	8	6	0
1	198	1353	40	7011	45	6975	119	757	6	41	18	10
2	251	726	78	583	45	994	121	525	0	35	22	1
3–4	525	813	168	93	68	368	204	534	0	111	40	0
5–9	492	686	202	32	137	47	268	496	3	172	70	0
10–14	96	149	88	1	38	2	45	85	1	63	27	0
15–24	33	51	54	2	13	1	14	34	2	47	5	0
25+	12	24	34	5	12	0	9	21	0	24	5	0
Total	1626	4187	666	7882	363	8707	804	2553	12	501	193	11

### Tree model

**Fig. 8:**
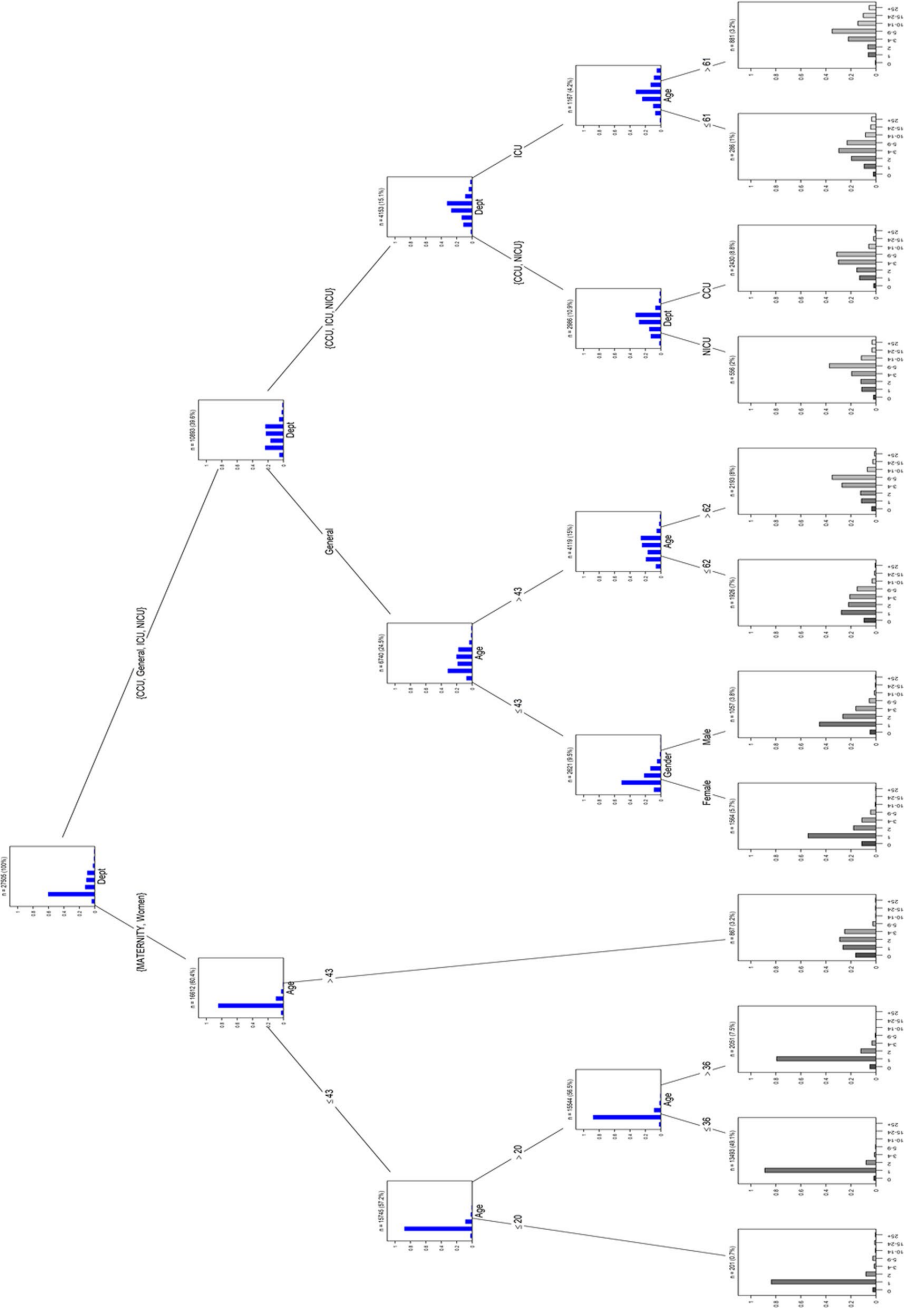
Tree plot that demonstrates the dispersion of patients in the study

The tree model contained 12 end nodes or clusters. For each end node, the categorized response variable’s bar chart is plotted, and the probabilities of categories are given. Due to the tree plot (Fig. 8), there was a different LOS pattern between Maternity-Women and the CCU-General-ICU-NICU ward groups. There was a significant difference in LOS between patients > 43 and ≤ 43 years old in the maternity-woman group. Patients under 43 years old typically had LOS of just one day, while those over 43 years old typically had LOS between 1 and 4 days. Moreover, the ≤ 43 years old group had two additional distinction points: 20 and 36 years. CCU-General-ICU-NICU branch was split into two different groups. The general ward was separated from the other three wards (CCU, ICU, and NICU). The majority of patients in the general ward had LOS between 1 and 9 days, but one day was more typical during this time. Similar to the Maternity-Women branch, patients in the general ward were divided by age with a cutoff of 43 years. There was a distinction between the genders who stayed in the general ward. Female patients were more likely than males to be discharged from the hospital in both cases of less than one day and one day of LOS. However, there were more male patients than female patients who had LOS of 2–4 days. There was a significant difference between inpatients aged > 62 and ≤ 62 years in the General ward. Those younger than 62 years in this ward had LOS of one day, two days, 3–4 days, and 5–9 days. But the majority of patients older than 62 years were in the 5–9 group. In the general ward, older patients required more days of hospitalization. In the CCU-ICU-NICU branch, patients usually had LOS between 3 and 9 days. In this branch, the ICU was separated from two other wards. Patients over 61 years old stayed longer in the ICU than patients under 61. Patients in the NICU and CCU typically had LOS of 5 to 9 days (Fig. 8).

## Discussion

In hospitals, there are major performance indicators, including LOS, bed occupancy rate, bed turnover, bed turnover interval, and mortality rates that should be determined and monitored regularly [15]. This study aimed to evaluate each inpatient’s LOS and its associated factors in six different wards of the hospital using statistical analysis along with data mining by the R-studio program. The average LOS in the study population was about two days. Results also demonstrated that about 60% of patients were discharged after one night of hospitalization. According to reports, hospitals in Iran had an average LOS of 3.6 days in 2017 [16]. According to 2019 statistics, the mean LOS in hospitals of Economic Co-operation and Development (OECD) member countries varies between 4.1 days in Turkey and 16 days in Japan [17]. A systematic review and meta-analysis on 13 studies from Iranian hospitals published between March 2006 and April 2017 reported that the rate of inappropriate and unnecessary LOS was 4.2% (95% Cl: 1.8–9.4%). This value was reported to be 22% in Turkey, 18.7% in Spain, 37% in Italy, and 14.9% in South Korea [16]. A decrease in hospital performance and productivity, a rise in hospital-acquired infections, and higher costs to the health system can all result from inappropriate and unnecessary LOS.

According to the literature, LOS can be impacted by a variety of factors [18]. These include age, race, place of birth, type of disease and primary diagnosis, hospital size, hospital location, the day of hospital admission, hospitalization ward, complications, hospital infections, diagnostic and therapeutic procedures for patients and their frequency, delayed tests, imaging, and surgery, the admitting physician’s specialty as well as some comorbidities [15]. Regarding age, we observed that the older patient had longer LOS than the younger. Interestingly, patients older than 43 years old had longer LOS. This could be partially attributed to women’s menopausal period. Additionally, patients’ LOS patterns varied between the ages of 20 and 36; this range may be related to women’s reproductive ages. These findings are generally consistent with the findings of other studies that older age is a risk factor for longer LOS [19–21]. In contrast, at least one study on patients with anxiety disorders in Iran found that older patients had a 9% shorter hospital LOS than younger patients (*P* = 0.036) [22]. Another research showed no significant relationship between age and LOS at a children’s hospital in southern Iran [23].

Women’s LOS patterns differed from men’s in terms of age. However, when the Maternity and Woman wards were excluded from the analysis, women’s LOS patterns in the other four wards were similar to men’s (Figs. 1 and 2). A study of factors related to patients’ LOS in a general hospital in southern Iran by using data mining techniques demonstrated that there was no significant association between gender and LOS [15]. Similar findings have been reported from a children’s hospital in the south of Iran [23].

In contrast, based on the analysis of the United States population database between 2002 and 2011, females had significantly greater hospital LOS for all combined procedures after shoulder arthroplasty than males. The authors attributed this difference to the fact that females were proportionally older and diagnosed with fractures at higher rates than males. Although reaching statistically significant, the clinical and economic impact of this difference (only 0.3 days) may not be significant [24]. Different clinical settings, health care systems, sample sizes, and disproportional distribution of certain diseases between males and females can lead to these various results regarding gender impact on LOS.

The results showed that patients with more severe diseases hospitalized in critical care wards had more LOS than those staying in other wards. In line with this finding, a study in 9 Dutch hospitals demonstrated that long-term LOS was associated with the disease’s severity, surgical operation complication, and restricted antibiotic use [25]. By using a statistical and data mining approach based on electronic health records at a tertiary general university hospital in South Korea, it has been demonstrated that disease severity significantly correlated with LOS [1]. According to the results of another study at a general hospital in southern Iran that exploited data mining techniques, it has been identified that the number of specialist consultations and number of para-clinical services had the highest weights of effect on LOS in hospitals. The authors stated that these two factors could be considered indexes of disease complexity as well as severity [15].

The main strength of the current study is assessing the possible association between demographic and clinical characteristics of patients and LOS in the hospital using the data mining technique by the R-studio program. The sample size was also remarkable. Nevertheless, due to a number of limitations, it seems prudent to interpret our results cautiously. The major one is not taking into account many other factors mentioned above like ethnicity, place of birth, type of disease and primary diagnosis, hospital size, hospital location, the day of hospital admission, complications, hospital infections, diagnostic and therapeutic procedures for patients and their frequency, delayed tests, imaging, and surgery, the admitting physician’s specialty as well as some comorbidities that may affect LOS in hospitals. Besides this, we did not determine the disease severity of patients hospitalized in ICU (e.g., SOFA or APACHE-II scores).

## Conclusion

The average LOS at the hospital in this study population was about two days. More than half of the patients were discharged after one night of hospitalization. The older patients had longer hospital LOS than the younger patients. By excluding Maternity and Woman wards, LOS in the hospital was comparable between males and females. Patients hospitalized in critical care wards with more severe diseases had more LOS than those in non-critical care wards. Regular and continuous evaluation as well as analyzing the LOS deem crucial for health policymakers and hospital managers to determine priorities, improve services, and manage resources more appropriately in hospitals.

## Data Availability

The datasets used and/or analyzed during the current study are available from the corresponding author on reasonable request.

## Abbreviations

LOS: Length of stay
CCU: Critical care unit
ICU: Intensive care unit
NICU: Newborn Intensive Care Unit
HIS: Hospital information system
SD: Standard deviation
SOFA: Sequential organ failure assessment
APACHE-II: Acute Physiology and Chronic Health Evaluation
AIC: Akaike information criterion
BIC: Bayesian information criterion
